# Stomal stenosis during gradual closure of a traumatic abdominal wall hernia

**DOI:** 10.1016/j.ijscr.2019.06.020

**Published:** 2019-06-16

**Authors:** Mitsuhiro Suzuki, Hisao Matsushima, Katsuki Uehara, Tatsuhiko Saiki, Atsuki Hayamizu, Toshirou Kamisasanuki, Daisuke Sugiki

**Affiliations:** Department of Emergency and Critical Care Medicine, Dokkyo Medical University Saitama Medical Center, Koshigaya, Saitama, Japan

**Keywords:** Abdominal wall reconstruction, Case report, Ileostomy, Negative pressure wound therapy, Stomal stenosis, Traumatic abdominal wall hernia

## Abstract

**Introduction:**

Traumatic abdominal wall hernias are often accompanied by intra-abdominal injuries, and a stoma may be required. Although rare, stomal stenosis can develop after the repair of a traumatic abdominal wall hernia.

**Presentation of case:**

A 65-year-old woman was in a head-on collision with a truck and was brought by ambulance to our facility. The findings of a physical examination and computed tomography scan suggested bowel perforation for which exploratory surgery was performed. The lacerated small bowel and sigmoid colon were resected and an ileostomy and colostomy were created. Abdominal wall reconstruction was impossible because of the large defect size. Repair of the abdominal wall was achieved by gradual closure of the fascia after surgery in combination with negative pressure wound therapy. Stenosis of the ileostomy occurred during this process and was surgically repaired.

**Discussion:**

We reconstructed the abdominal wall using negative pressure wound therapy in combination with sutures while minimizing the risk of abdominal compartment syndrome. This approach did not increase the intra-abdominal pressure, but it deformed the abdominal wall, resulting in unexpected stenosis of the ostomy.

**Conclusion:**

Gradual postoperative closure of a traumatic abdominal wall hernia with an ostomy in place may result in stomal stenosis. Stomal patency must be carefully evaluated during this process.

## Highlights

• Stomal stenosis can develop after repair of a traumatic abdominal wall hernia.
• Abdominal wall repair with negative-pressure wound therapy may be effective.
• Stomal patency must be evaluated during the abdominal wall hernia repair.

## 1 Introduction

Traumatic abdominal wall hernias are uncommon and defined as “herniation through disrupted musculature and fascia associated with adequate trauma but without skin penetration or evidence of a prior hernia defect at the injury site” [1]. The most common mechanism of injury leading to traumatic abdominal wall hernia is a motor vehicle collision. Netto et al. reported that most traumatic abdominal wall hernias in their series were in the lumbar triangle, where a seat belt would transmit force from an abrupt deceleration. They also concluded that all patients with an anterior traumatic abdominal wall hernia evident on clinical examination had associated intra-abdominal injuries requiring surgical intervention [2].

Many patients with traumatic abdominal wall hernias require abdominal wall reconstruction. An emergency laparotomy is indicated for hemostasis, the treatment of hollow viscus injuries, and the prevention of incarceration rather than hernial repair. Abdominal wall reconstruction is important because abdominal wall defects negatively impact patients. An ostomy may also be necessary in some cases of traumatic abdominal wall hernia, but stomal stenosis is rare during repair of traumatic abdominal wall hernia.

This work has been reported in line with the SCARE criteria [3].

## 2 Presentation of a case

**Fig. 1:**
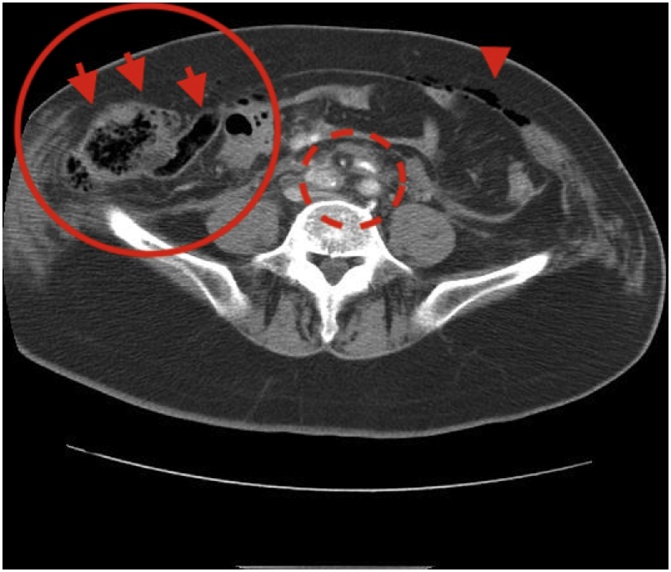
Enhanced computed tomography scan on admission.The scan revealed a defect in the abdominal wall (circle), herniation of the cecum and small bowel loops (arrow), free air in the abdomen (arrowheads), and an acute traumatic dissection of the abdominal aorta and common iliac artery (dotted circle).

A 65-year-old woman was the driver in a frontal collision with a truck at 50 km/h. She was alone in the car and wearing a seatbelt, and her car’s airbags deployed. She was transported to our facility by ambulance. On arrival, tachypnea and tachycardia with a pulse generally over 100 beats per minute occurred during resuscitation. The systolic blood pressure was maintained at greater than 100 mmHg. During fluid resuscitation, a physical examination showed tenderness and distension in the right lower quadrant with a seat belt sign. Her left leg was deformed with perforation of the skin, suggesting an open fracture of the tibia. She remained hemodynamically stable during the resuscitation. An enhanced computed tomography scan of the abdomen showed a defect of the abdominal wall, herniation of the cecum and small bowel loops, free air in the abdomen, and acute traumatic dissection of the abdominal aorta and common iliac arteries (Fig. 1).

She was urgently brought to surgery and the abdomen was explored through a long midline incision. The exploration revealed a 12 × 30 cm defect of the abdominal wall in the right lower quadrant, laceration of the ileum mesenteric injury with bowel ischemia, a transected sigmoid colon with extensive spillage, and a transected inferior mesenteric artery. The abdominal aorta was exposed distal to the inferior mesenteric artery, but no aortic bleeding was seen. The inferior vena cava and right ureter were also completely exposed at the same level. The abdominal wall defect was present along the iliac crest, and bleeding was noted at that site.

After achieving hemostasis, approximately 160 cm of ileum and lacerated sigmoid colon was resected. Due to extensive spillage of stool and vascular injuries that would possibly require graft repair, we performed an end ileostomy as well as an end colostomy to minimize the chance of any subsequent anastomotic leakage. The distal ends of the bowel were closed and placed in the abdomen.

The abdominal wall could not be closed at the initial operation due to the large defect size. Negative pressure wound therapy was thought to be useful for temporary coverage. We could not perform a tension-free repair because of the risk of infection with extensive intra-abdominal contamination. The fascial defect was closed as much as possible and negative pressure wound therapy instituted for the part of the defect that could not be definitively closed. At the end of the operation, the patency of both stomas was verified digitally.

**Fig. 2:**
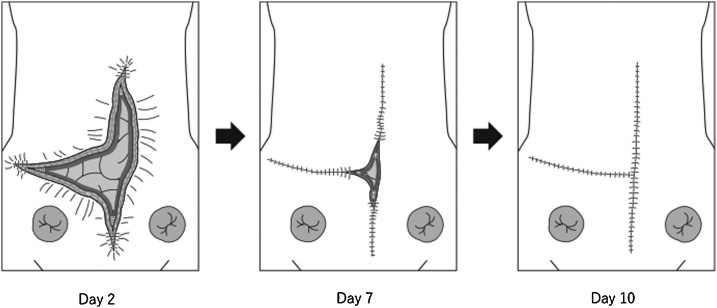
Illustrations of procedures for abdominal wall closure.Abdominal wall hernia repair was performed with a negative pressure wound therapy devices (not shown). On postoperative day 2, when removing the device, the abdominal wall appeared lax and several sutures were placed in the fascia. After repeating this process daily, the abdominal wall was completely closed by day 10.

The following day, we removed the negative pressure wound therapy devices and found that the abdominal wall could be approximated; sutures were placed at the bedside. After repeating this technique, the abdominal wall was completely closed by postoperative day 10. A series of these procedures is shown in Fig. 2. During this gradual closure, there was no evidence of abdominal compartment syndrome (as measured by bladder pressure and clinical status) or respiratory complications.

**Fig. 3:**
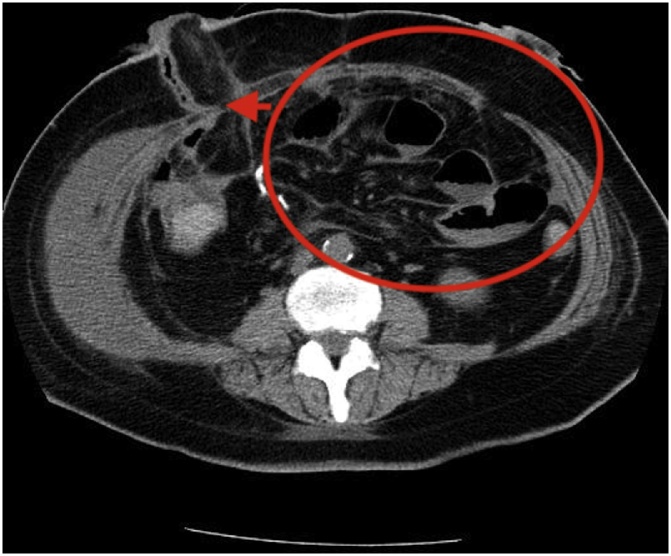
Computed tomography scan 16 days after ileostomy and colostomy.The patient complained of nausea and vomited on day 16. Computed tomography scan revealed dilation of the small bowel (circle) and angulation in the abdominal wall impinging on the ileostomy (arrow).

Non-operative management to dissection of the abdominal aorta and common iliac arteries was selected because the patient had no symptoms of ischemia or bleeding. On postoperative day 16, the patient complained of nausea and vomiting. A computed tomography scan of the abdomen showed that dilation of the entire small bowel and angulation of the abdominal wall impinging on the ileostomy (Fig. 3). A digital exam of both stomas was impossible because of stenosis at the level of the fascia. On postoperative day 19, the fascia was incised in the operating room with the patient under general anesthesia and the tissue around the ileostomy was opened to release the stenosis at the site of the ileostomy, with clinical improvement.

There were no subsequent obstructions at that site. The colon proximal to the end colostomy was not dilated and the colostomy was observed. No symptoms developed at the colostomy site. Closure of the ileostomy and a colostomy were performed 14 months after the initial exploration; there was no evidence of hernia recurrence at 2 years follow-up, and the patient had no complaint about reconstructed abdominal wall.

## 3 Discussion

Traumatic abdominal wall hernia repair with concurrent injuries to the intra-abdominal organs is a major surgical challenge, especially in the presence of massive fecal contamination. Biologic mesh is being used to repair abdominal wall defects in a contaminated field [4]. In a study of ventral hernia repairs with biologic mesh, Boules et al found rates of hernia recurrence and postoperative complications of 34.9% and 39%, respectively [5]. These rates are unacceptably high for the repair of a traumatic abdominal wall hernia. Significantly, biologic mesh is not currently available in Japan. Composite polypropylene mesh with absorbable collagen film is used in Japan, but this was inappropriate in the present patient case due to massive contamination. Therefore, we performed the hernia repair without using a tension-free technique.

Negative pressure wound therapy allows adequate drainage of peritoneal fluid, decreases the risk of abdominal compartment syndrome, and maintains perfusion of intra-abdominal organs [6]. van Hensbroek et al. found that negative pressure wound therapy is associated with high fascial closure rates and low mortality rates [7], which suggests that negative pressure wound therapy reduces dehiscence at the fascial edge and makes it possible to perform primary fascial closure. We concluded that we could reconstruct the abdominal wall using negative pressure wound therapy in combination with sutures while minimizing the risk of abdominal compartment syndrome. This approach did not increase the intra-abdominal pressure, but it did deform the abdominal wall, resulting in unexpected stenosis of the ostomy.

## 4 Conclusion

In the present patient, it was possible to reconstruct the abdominal wall without using a tension-free technique and without hernial recurrence. Abdominal wall reconstruction by gradual suturing along with negative pressure wound therapy in a contaminated field may be effective. This case report demonstrates the utility of this technique and describes a rare complication of stomal stenosis that may occur during the repair of traumatic abdominal wall hernia with an ostomy. Surgeons must be aware of this possible complication when repairing abdominal wall hernias.

## Funding

No funding was received.

## Ethical approval

This study is exempt from ethical approval in our institution.

## Consent

Written informed consent was obtained from the patient for the publication of this report.

## Author contribution

Mitsuhiro Suzuki: treated the patient and conducted operations, collected the data, and wrote the manuscript.

Hisao Matsushima: prepared the manuscript.

Katsuki Uehara: conducted the examination on this patient, treated the patient, and conducted operations.

Tatsuhiko Saiki: helped treat the patient.

Atsuki Hayamizu: helped treat the patient.

Toshirou Kamisasanuki: helped treat the patient.

Daisuke Sugiki: treated and conducted operations.

All authors approved submission of the final article.

## Registration of research studies

This case report does not require registration as a research study.

## Guarantor

Dr Mitsuhiro Suzuki is the guarantor of this case report.

## Provenance and peer review

Not commissioned, externally peer-reviewed.

## Declaration of Competing Interest

The authors declare no conflicts of interest for this article.
